# In vitro and in vivo low-dose exposure of simulated cooking oil fumes to assess adverse biological effects

**DOI:** 10.1038/s41598-022-19558-x

**Published:** 2022-09-20

**Authors:** Shuangde Li, Li Wang, Shanyue Guan, Shuyun Zhou, Yunfa Chen

**Affiliations:** 1 State Key Laboratory of Multiphase Complex Systems, Institute of Process Engineering, Chinese Academy of Sciences, Beijing, 100190 China; 2 Key Laboratory of Photochemical Conversion and Optoelectronic Materials Technical, Institute of Physics and Chemistry, Chinese Academy of Sciences, Beijing, 100190 China; 3 University of Chinese Academy of Sciences, Beijing, 100049 China

**Keywords:** Biochemistry, Cell biology

## Abstract

Cooking oil fumes (COFs) represent a major indoor environmental pollutant and exhibit potent mutagenic or carcinogenic health effects caused by containing various heterocyclic aromatic amines (HAAs) and long-chain aldehydes. Despite some evaluation of the cumulative exposure of COFs to cancer cells under high concentration were evaluated, their biological adverse effects with low-dose exposure to healthy cells had been inadequately investigated. Herein, we firstly scrutinized the three selected typically toxic compounds of heterocyclic amine 2-amino-1-methyl-6-phenylimidazo[4,5-b]pyridine (PhIP), 3,8-dimethylammidazo[4,5-f]quinoxalin-2-amine (MeIQx) and trans, trans-2,4-decadienal (TDA)) emitted from COFs. In vitro studies revealed that the PhIP, MeIQx and TDA aerosol particles were negligible toxicity to cancer cells (A549 and HepG-2) but strong cytotoxicity to normal healthy cells (HelF and L02) under 0.5–4 μg/mL low dose exposure based on the reactive oxygen species (ROS) mechanism. In vivo studies demonstrated that PhIP caused significant lung and liver damage after exposure to PhIP for 30 days with mice. These results indicated the direct proof of healthy cell damage even at low-dose exposure to HAAs and aldehydes.

## Introduction

Household air pollution generated from cooking is severe and often involves fine particles (PM_2.5_), ultrafine particles (UFPs), and volatile organic compounds (VOCs) from the volatilization of oils, which have adverse impacts on the environment and human health^1–8^. There are widely investigated and ample accepted relationships between exposure to COFs and mutagenic/carcinogenic diseases^9^, such as chronic bronchitis^10^ and lung cancer^11–13^, through statistical analysis and epidemiological studies from randomly selected female nonsmokers^14–16^. Furthermore, cooking habits (cooking methods and oil use) are related to the risk of lung cancer^17^. However, long-term use of a fume extractor in cooking can reduce the risk of lung cancer by approximately 50%^2^. Regarding the toxicity and exposure of COFs, current studies have focused on the typical emission components, polycyclic aromatic hydrocarbons (PAHs), heterocyclic aromatic amines (HAAs) and long-chain aldehydes, and their health risk assessment.

The concentration and particle size distribution of PAHs emitted by thermal cooking vary regardless of food type, with the main components being 3- and 4-ring PAHs^18^, which are associated with lung diseases^19^ and higher risks for preschool-aged children^20^. PAHs derived from barbecue fumes were reported to show a more important pathway for dermal intake than inhalation^21,22^. Heterocyclic amine 2-amino-1-methyl-6-phenylimidazo[4,5-b]pyridine (PhIP) and 3,8-dimethylamidazo[4,5-f]quinoxalin-2-amine (MeIQx) were representative HAAs during meat cooking at high temperatures due to the Maillard reaction^23^. HAAs were known to form DNA adducts or HAA excretion governed by the metabolic pathway, which leaded to an increase in human cancer risk from dietary exposure^23^. The PhIP concentrations are between 1 and 70 ng/g fried fish varied by pan-broiled and over-cooked at 200 °C and barbecued at 270 °C, MeIQx up to 23 ng/g meat^23^, and even 268.1 ng/g cooking aerosols through Chinese dishes of frying fish in a short period^24^. The neurotoxic effects of PhIP were first observed from rat embryos in vitro to dopaminergic neurons due to oxidative stresses^25^. An amount of 0.25 ng MeIQx per 1 g of meat per min was estimated based on the mutagenic response^24^. MeIQx proved carcinogenicity in mice fed a diet containing 0.06% MeIQx for 84 weeks, which induced liver tumors in 43% of males and 91% of females^26^. Long-chain trans, trans-2,4-decadienal (TDA), the most prevalent aldehydes occupied 12.2–34.2% of total aldehyde concentrations among 706–2482 μg/m^3^ in COFs produced while cooking pork loin, varying from cooking method, cooking oil, and food type^27,28^, which drew great attention because they are considered carcinogenic^15,29^.

Although there had already been some scientific research on the association of cooking oil fume exposure with health risk, it had mainly focused on cancer cell culture under higher COF concentrations or higher doses of characteristic toxic substances in vitro; in addition, high COF exposure or diet inhalation had been used to detect body weight in rats in vivo. However, there were absent appropriate test protocols or relevant data for the biological effects of low dose exposure to specific and characteristic aerosol particles but not COFs in healthy and cancer cells for comparison. Furthermore, HAAs, such as PhIP or TDA, mainly existed with aerosols, which might be incorporated in the oil mist as well as in carbonaceous particles^23^. Recently, Poudel investigated whether PhIP and PhIP@OA particles showed less cytotoxic effects on SHSY5Y, MRC5, and human dermal fibroblast cells than dissolved PhIP but clearly induced premature senescence activities with concentrations up to 100 μg/mL^30^, which resulted in remarkable differences between the dissolved and particulate forms of PhIP in cytotoxic profiles. There is still a need for more comprehensive studies with infinitely close to real fume, which is crucial for accurate estimation of their impacts on human health through not only widely studied lung cells but also liver cells and rats in vitro and in vivo.

**Figure 1:**
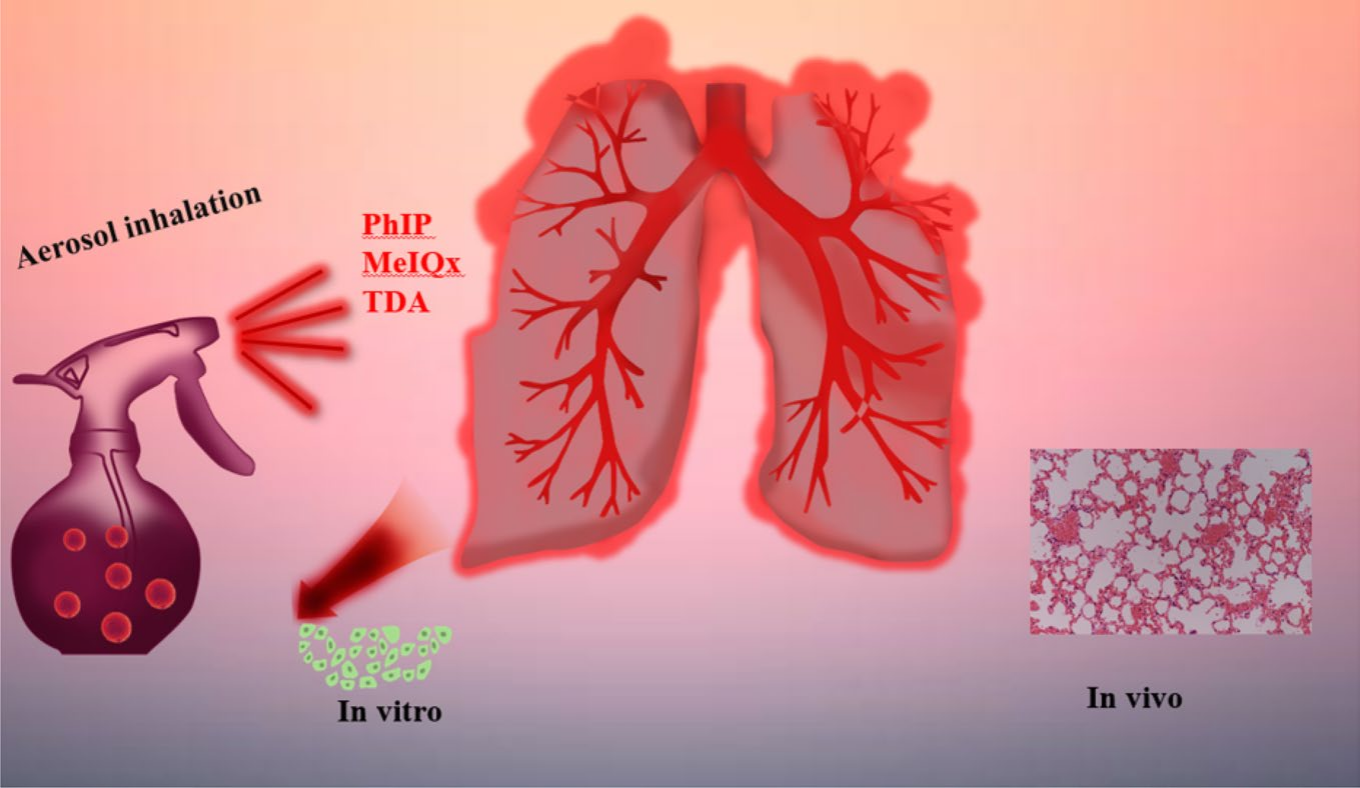
Schematic illustration of the evaluation of PhIP, MeIQx and TDA aerosols exposure on the HepG-2, L02, HelF and A549 cells in vitro and on the mice in vivo.

Herein, for the first time, three typical hazardous chemicals from COFs, PhIP, MeIQx and TDA, were chosen as human carcinogenic or mutagenic compounds to systematically evaluate the damage to both cancer and healthy cells with low dose exposure. Schematic illustration of the evaluation of those aerosols exposure on the HepG-2, L02, HelF and A549 cells in vitro and on the mice in vivo were shown in Fig. 1. Thus, the ultimate goal of this study was to identify the biological adverse effects of low dose exposure. PhIP caused much more damage to healthy liver and lung cells than MeIQx and TDA. The results might guide reasonable cooking methods with less emission of characterized compounds, such as PhIP, thus with less carcinogenic or mutagenic risk.

## Results

### Physicochemical characterizations of aerosol particles

**Figure 2:**
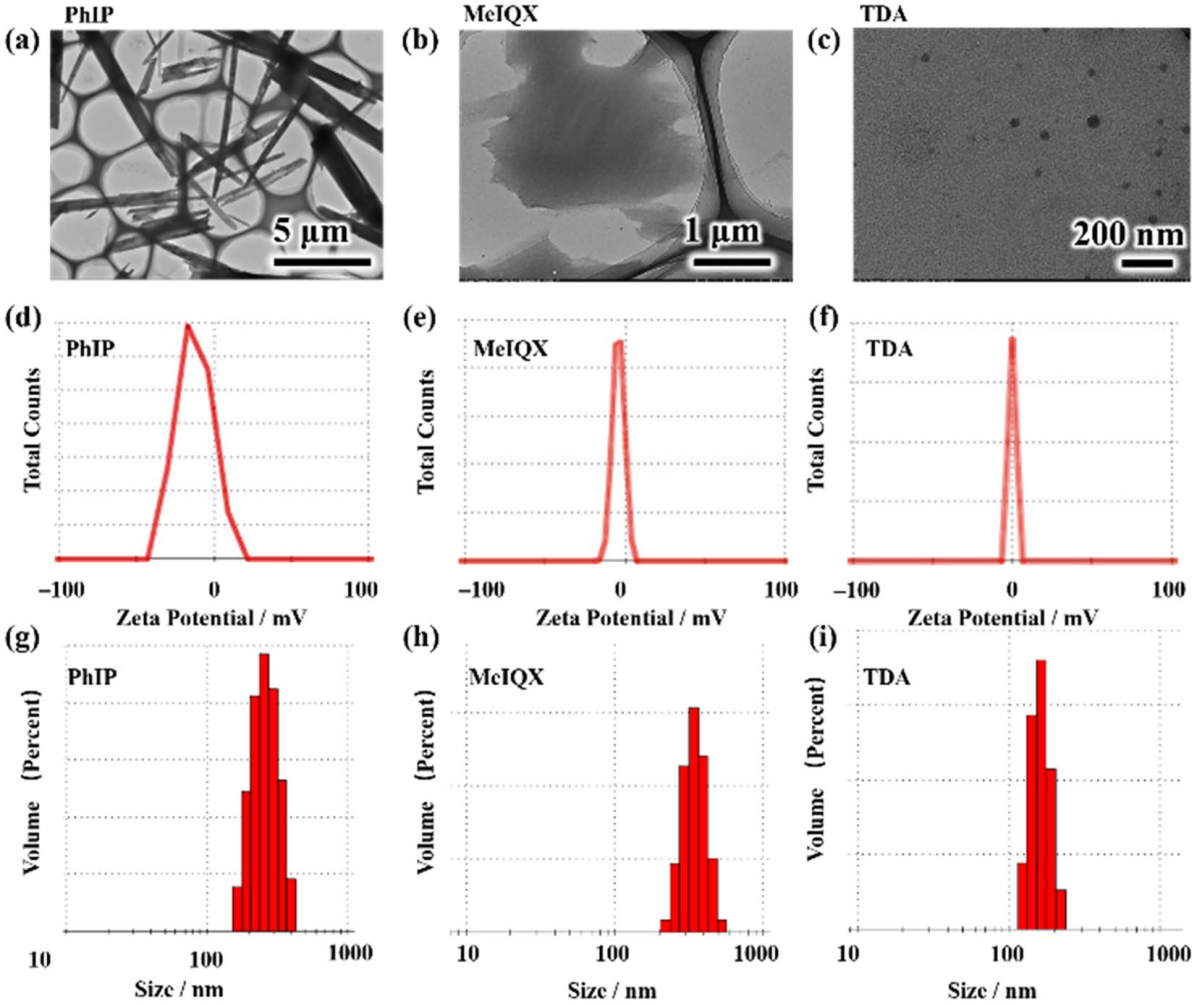
TEM (**a**–**c**) images, Zeta potentials (**d**–**f**) and (**g**–**i**) Dynamic Light Scattering (DLS) size distribution of PhIP, MeIQx and TDA aerosol particles, respectively.

The PhIP, MeIQx and TDA aerosol particle samples were synthesized via an easy method through a compressor nebulizer, and their morphology and size were investigated using TEM measurements, as shown in Fig. 2a–c. PhIP was rod-like with a uniform particle size of 5 ± 0.6 μm, and MeIQx revealed a strip-like morphology with a size of 1.5 ± 0.2 μm. In contrast, TDA presents an irregular round morphology with a size of 20–40 nm. PhIP particles obtained by collison atomizer with 90 mg PhIP dissolved in 100 mL DMSO exhibited anisotropic irregular morphologies with a size of 71 ± 5.4 nm, some of those with rods shape with 200 nm in length^30^. The increased PhIP rod size observed in our system might due to the PhIP aerosols obtained by a compressor nebulizer with higher concentration by dissolved 10 mg PhIP in 1 mL DMSO. The rod-like PhIP particles might be caused by aerosolization, while the dissolved PhIP in DMSO were in the existence of solute ion state^30^. Furthermore, the zeta potentials of PhIP, MeIQx and TDA were investigated in the dispersion solution (Fig. 2d–f). The obtained PhIP particles were negatively charged with a zeta potential of − 11.7 mV, while MeIQx and TDA with − 5.6 and − 0.2 mV, respectively, indicating that PhIP was more stable in the dispersion solution than MeIQx and TDA. In addition, the particle sizes of PhIP, MeIQx and TDA were determined by dynamic light scattering (DLS) to be 588 nm, 623 nm and 162.1 nm (Fig. 2g–i), respectively. Both the TEM and DLS results exhibited the fabrication of aerosol particles for the evaluation of their adverse effects.

### In vitro cytotoxicity with CCK-8 assay

**Figure 3:**
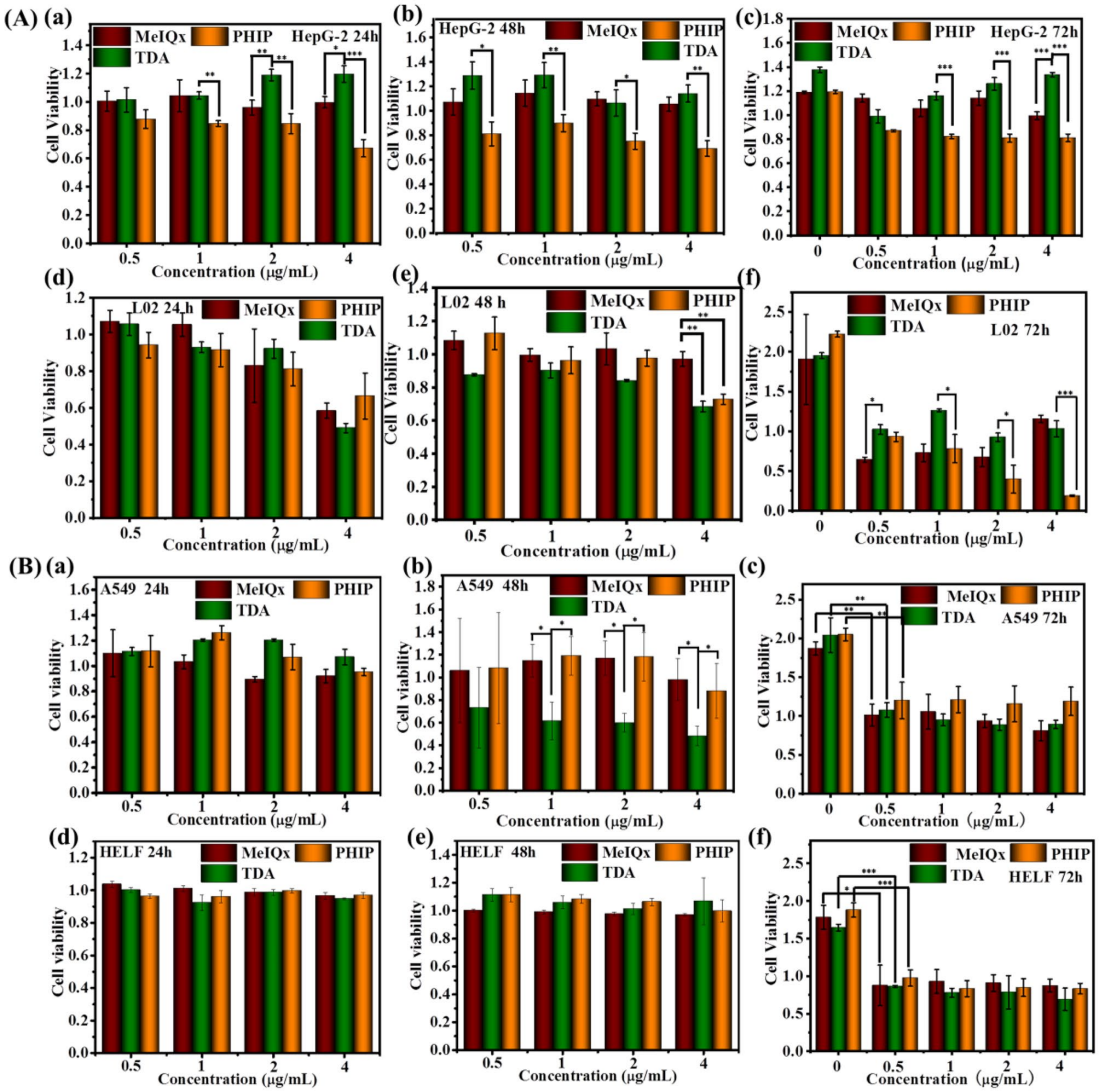
CCK-8 results for (**A**) L02 cells and HepG-2 cells and (**B**) A549 cells and HelF cells incubated with PhIP, MeIQx and TDA under varied concentration from 0.5 to 4 μg/mL, 200 μL, for 24 h, 48 h and 72 h, respectively. The results were given as a mean value of three independent experiments ± SD. The statistical analysis was performed by the one-way analysis of variance-ANOVA followed by Turkey’s post hoc test (*p < 0.05, **p < 0.01, ***p < 0.001).

**Figure 4:**
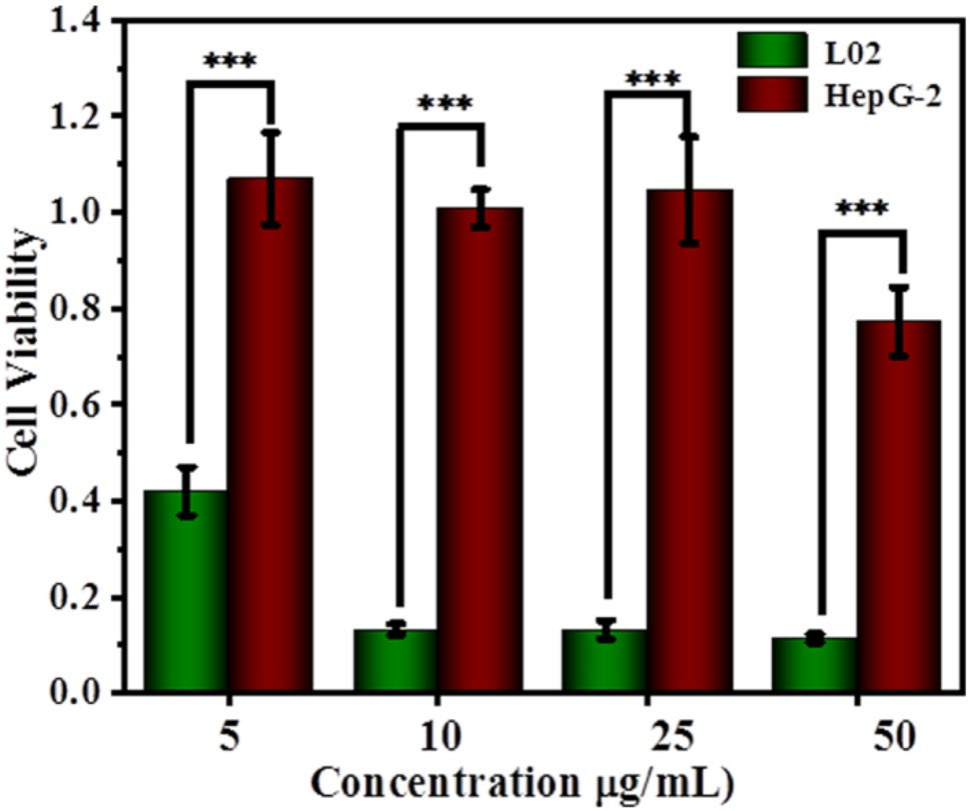
CCK8 results about the HepG-2 and L02 cells incubated with PhIP aerosols for 24 h with varied concentration from 5 to 50 μg/mL, 200 μL, respectively. The results were given as a mean value of three independent experiments ± SD. The statistical analysis was performed by the one-way analysis of variance-ANOVA followed by Turkey’s post hoc test (*p < 0.05, **p < 0.01, ***p < 0.001).

To assess the toxicity of PhIP, MeIQx and TDA aerosol particles in tumor and normal cells, the standard Cell Counting Kit-8 (CCK-8) assay was applied. The control experiment were carried out for HelF, HepG-2, L02 and A549 cells incubated with the DMEM solution under the absence of PhIP, MeIQx and TDA for 24 h, 48 h and 72 h, and their CCK-8 results exhibited the absence of cell apoptosis with around 100% cell viability (without shown here). The cell viability of Y-axis in Fig. 3 indicated relative cell viability with the control group under DMEM incubation for consistent conditions. Tumor cell HepG-2 and normal liver cell L02 were incubated with PhIP, MeIQx and TDA particles at various concentrations (from 0.5 to 4 μg/mL) for different times (24 h, 48 h, 72 h), and their cell viability results were shown in Fig. 3A. A significant toxicity effect occurred on L02 cells, which was enhanced gradually with increasing dosage from 0.5 to 4 μg/mL after the incubation time extended from 24 to 72 h for PhIP (Fig. 3Ad–f). In contrast, PhIP did not show remarkable cytotoxic effects on HepG2 cells, even at concentrations up to 4 μg/mL after 72 h incubation (Fig. 3Aa–c). As shown in Fig. 3A, MeIQx sample also presented cytotoxicity to L02 cells, while there was weak cell damage through TDA incubation. The half maximal inhibitory concentration (IC_50_) of PhIP was 1.77 μg/mL, which was less than that of TDA (552.1 μg/mL) and MeIQx (6.01 μg/mL). This could further support that PhIP did harm the liver even at small amounts, while L02 cells showed a strong tolerance to TDA. For further assessment the cell damage of PhIP to HepG-2 and L02 cells, they were incubated with PhIP particles at higher concentrations (from 5 to 50 μg/mL) for 24 h. The results displayed that the particulate PhIP did not show remarkable cytotoxic effects on the cancer liver cell lines HepG-2, even at concentration up to 50 μg/mL (Fig. 4a), while presented significant cell death on the normal cells L02 under same condition (Fig. 4b). It can be concluded that PhIP aerosol particles could damage normal liver cell with the exposure from low to high dosage.

Furthermore, to evaluate cytotoxicity to the lung, A549 and HelF cells were measured for cell cytotoxicity after incubation with PhIP, MeIQx and TDA aerosol particles at various concentrations for different times (Fig. 3Ba-f). All three samples exhibited negligible effects on the survival of both A549 and HelF cells, even up to 4 μg/mL concentrations for the 24 and 48 h incubation (Fig. 3Ba,b,d,e). After prolonging to 72 h incubation, there was weaker cell cytotoxicity for HelF cells rather than A549 cells (Fig. 3Bc,f). The results implied that the three typical chemicals of cooking fumes did not damage lung cells despite those chemicals directly breathing into the lung under low dose and less incubation time. Considering the above results, one could conclude that only low dose PhIP aerosol particles could harm the liver instead of the lung.

### In vitro cytotoxicity with flow cytometry and ROS formation

**Figure 5:**
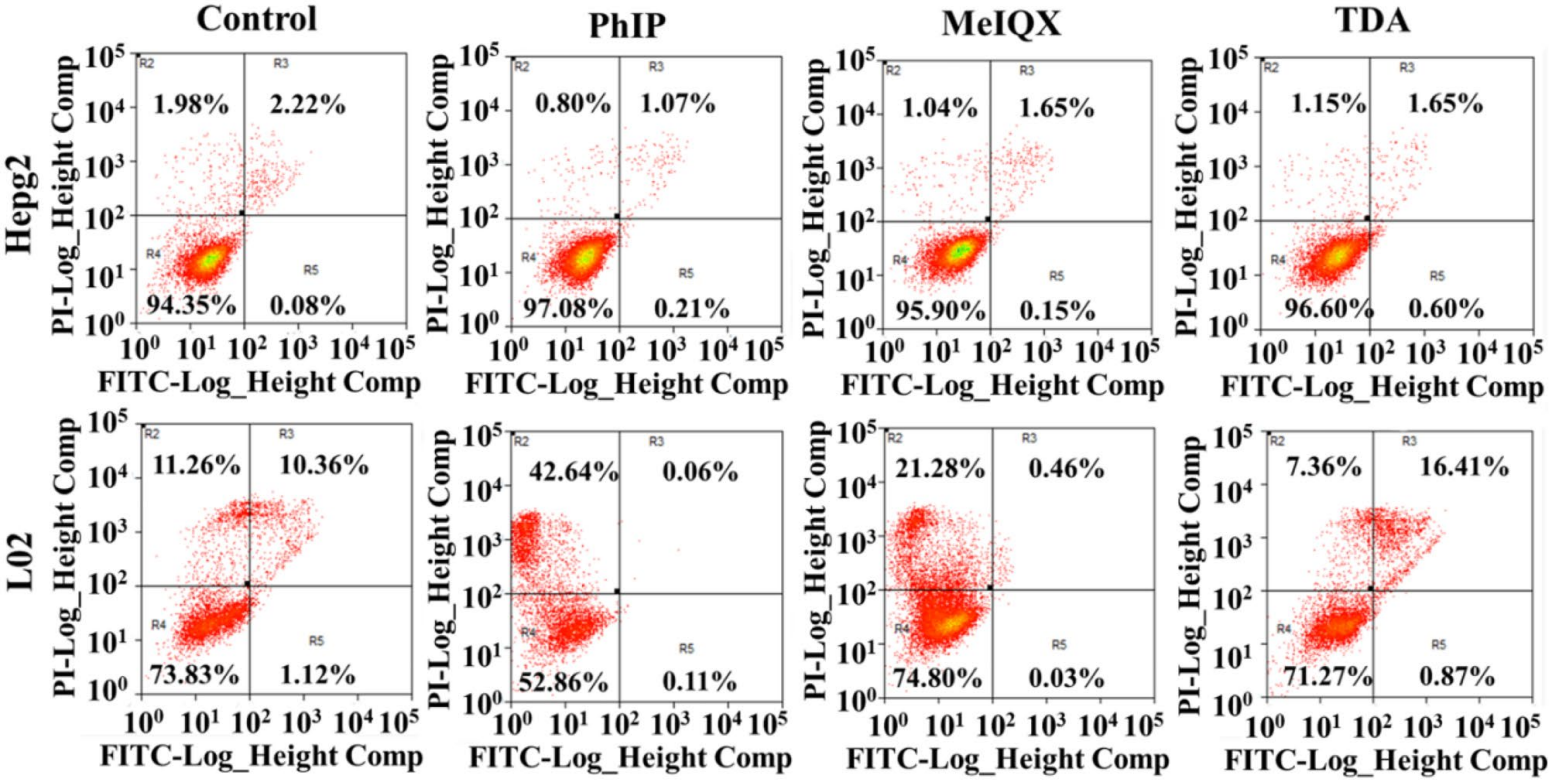
Flow cytometry of PhIP, MeIQx and TDA on HepG-2 and L02 cells after 4 μg/mL, 200 μL, incubation for 24 h. Regions R2, R3, R4 and R5 represent necrotic, late apoptotic, live and early apoptotic cells in the population.

Flow cytometry is a sophisticated instrument measuring multiple physical characteristics of a single cell, such as size and granularity, simultaneously as the cell flows in suspension. This approach makes flow cytometry a powerful tool for detailed analysis of complex populations in a short period of time^31^. The apoptosis (R3 and 5 regions) together with necrosis ratios (R2 region)^32^ were detected with flow cytometry for PhIP, MeIQx and TDA in HepG-2 and L02 cells and was shown in Fig. 5. The live ratios (Region 4) of PhIP, MeIQx and TDA on HepG2 cells were approximately 95.90–97.08%, in comparison with that of the control group at 94.35%, while the live ratios of PhIP on L02 cells at approximately 52.86% were significantly lower than those in the control group and MeIQx- and TDA-treated groups, with the values at 73.83%, 74.80% and 71.27%, respectively. The results indicated that the L02 cell proliferation was inhibited after treatment with PhIP. Furthermore, the increase in the percentage of R2 regions with PhIP and MeIQx treatment on L02 cells with 42.64% and 21.28% in comparison with 11.26% (control group) and 7.36% (TDA group), suggested that necrosis caused by PhIP and MeIQx treatment were progressing. Conversely, there were little effect on HepG2 cells exposed to PhIP, MeIQx and TDA. The results for the adverse effect of PhIP to L02 cells were almost identical to those of the CCK-8 results.

**Figure 6:**
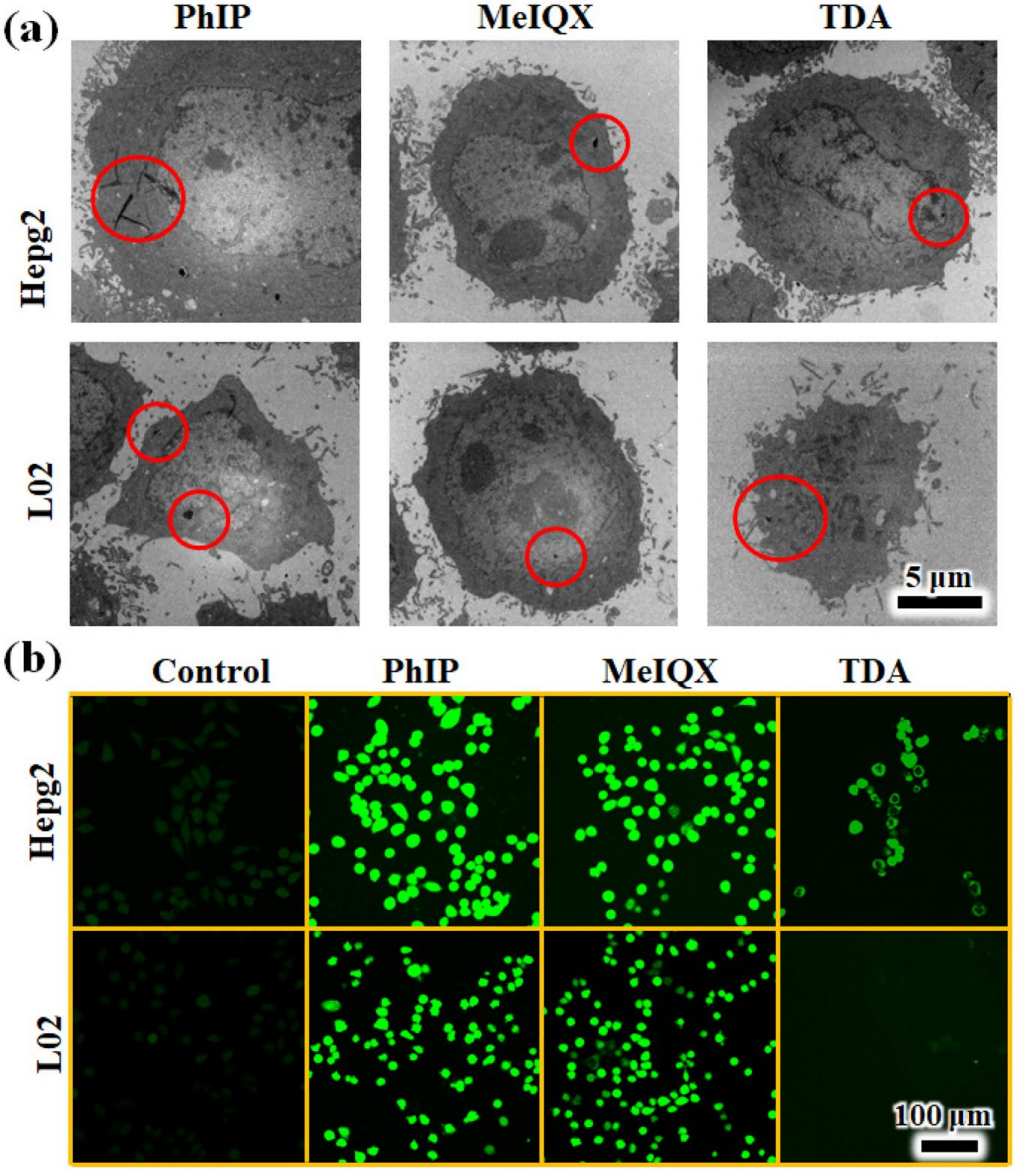
(**a**) TEM images of HepG-2 and L02 cells after 4 μg/mL, 200 μL, incubation for 24 h and (**b**) confocal imaging of HepG-2 and L02 cells stained with DCF-DA for the evaluation of ROS.

To investigate whether exposure to PhIP, MeIQx and TDA samples caused direct or indirect damage to the cellular tissues, cells were observed under TEM. PhIP, MeIQx and TDA can intrude into the cytoplasm with their initial particle size in HepG-2 cells, while PhIP and MeIQx can also be observed in the nucleus with reduced particles in L02 cells. This phenomenon indicated that exposure to PhIP and MeIQx resulted in L02 cell damage (Fig. 6a). To understand the mechanism of PhIP, MeIQx and TDA inducing cell apoptosis, we stained HepG-2 and L02 cells with DCF-DA to investigate the generation of ROS. Dichlorodihydrofluorescein diacetate (DCF-DA) has been widely used in detection of reactive oxygen species (ROS), which was commonly used as a standard to determine the toxicity of pollutants in the medical fields^33^. As revealed in Fig. 6b, no obvious green fluorescence intensity was found in the control group. In contrast, a strong green fluorescence signal was found in both PhIP and MeIQx samples, while a negligible green signal was detected after incubation with TDA. The data might deduce that PhIP and MeIQx aerosol particles bring ROS. ROS detected by a local region taken with confocal, could be used for qualitative analysis in relation with biological adverse effects. Combined with the results of flow cytometry in Fig. 5, the cytotoxicity of PhIP to L02 cells could be considered.

### In vivo toxicity with mice weight and organs staining

**Figure 7:**
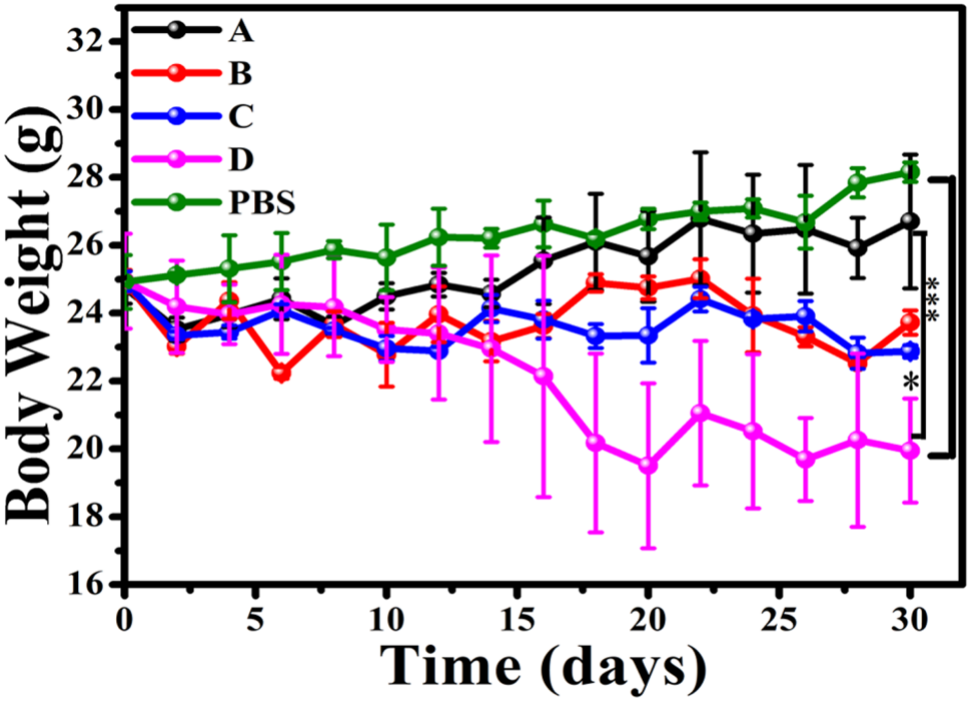
Body weights of mouse groups after various treatments of PhIP aerosol particles. A, B, C, D represent PhIP concentrations of 10, 20, 50, and 100 μg/mL, 10 mL. The results were given as a mean value of three independent experiments ± SD. The statistical analysis was performed by the one-way analysis of variance-ANOVA followed by Turkey’s post hoc test (*p < 0.05, **p < 0.01, ***p < 0.001).

To further assess the in vivo toxicity of PhIP, we mimicked the cooking environment and sprayed aerosols with various concentrations of PhIP (10, 20, 50, 100 μg/mL) onto male nude Balb/c mice. Considering that the cooking environment was a continuous accumulation process, it was essential to assess the damage to the mice within a period of time. The body weights of the mice were monitored every two days. As indicated in Fig. 7, no obvious body fluctuation was found in the control group or the A group (10 μg/mL). In contrast, the body weights of mice showed obvious decreases as the concentration increased from 10 to 20, 50 and 100 μg/mL with the incubation time lasting to 30 days, demonstrating the nonnegligible adverse consequence of the increasing dose of PhIP on mice (Fig. 7). Specifically, the body weights of mice decreased to 20.0 g for 100 μg/mL PhIP incubation under 30 days, in comparison with the increased gradually body weights to 27.8 g found in the control group.

**Figure 8:**
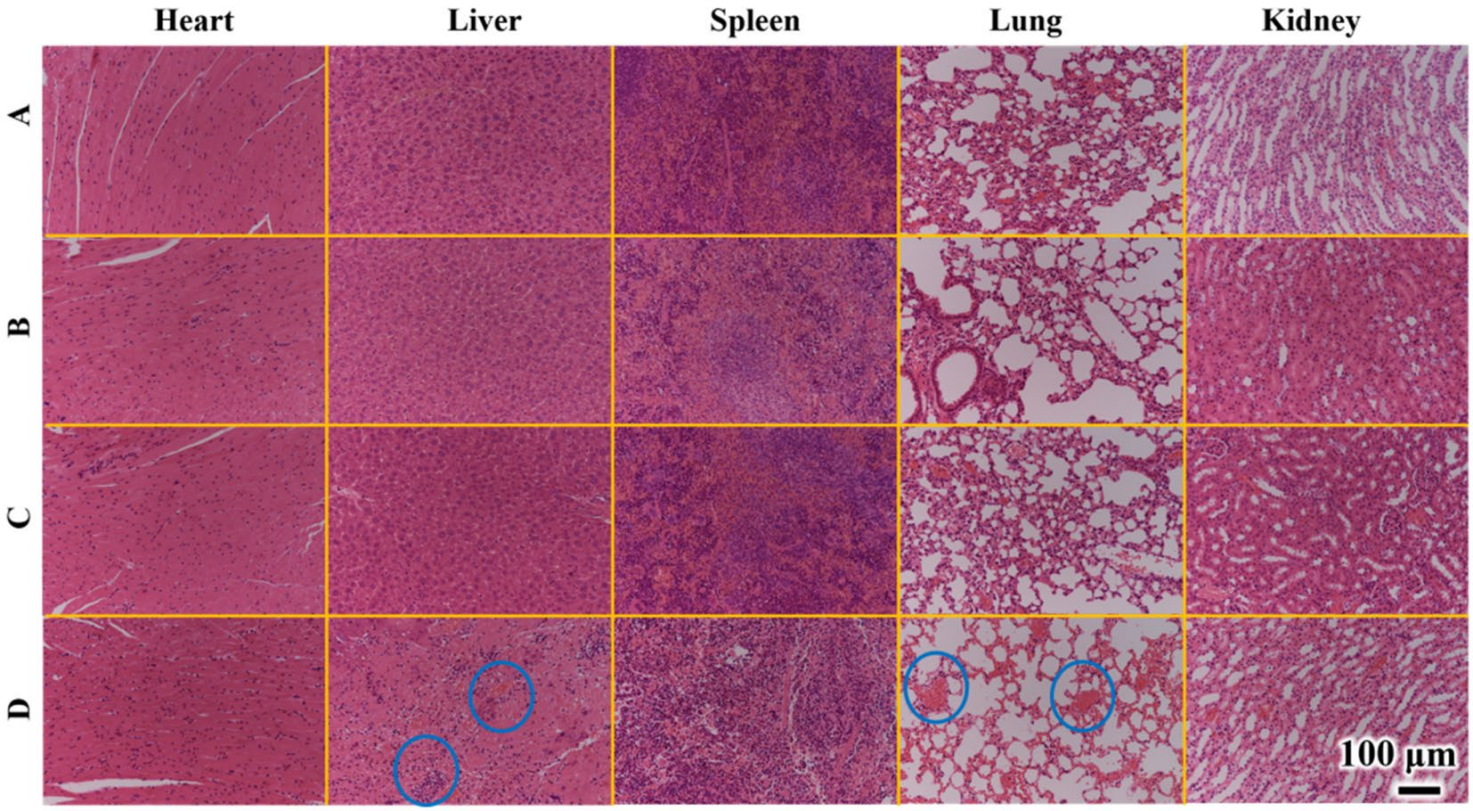
H&E-stained images of main organs (heart, liver, spleen, lung, kidney) from the different treatments PhIP (A, B, C, D represents 10, 20, 50, 100 μg/mL) for 30 days exposure of mice.

Additionally, hematoxylin and eosin (H&E) staining of major organ tissues further confirmed the influence of PhIP on mice^34^. As Fig. 8A-C shown, there was no obvious tissue damage or inflammation in major organs, including the heart, liver, lung, spleen and kidney, when the administration dosage was low (10 μg/mL), even up to 50 μg/mL. As expected, distinct inflammation in the liver and lung was observed once the concentration of PhIP increased to 100 μg/mL (Fig. 8D). However, the heart, spleen and kidney, might be less sensitive to PhIP exposure than the liver and lung, because no significant damage was observed in this study up to 100 μg/mL. These results confirmed that PhIP could cause irreversible damage to the liver and lung under a continuous accumulation process. The biological adverse effects from PhIP to mice tissues were consistent with the reduced body weight of mice.

## Discussion

Considerable progress has been made in research on biological adverse effects with the exposure of cooking emission species through cell incubation method over the last 20 years mainly focused on the high concentrations of PhIP, MeIQx and TDA solution. Most of the past studies focused on the cell cytotoxic effects through adding the PhIP compound dissolved in DMSO^35^. Poudel firstly reported that there existed remarkable differences between the dissolved and particulate forms of PhIP in cytotoxic profiles for SHSY5Y, MRC5, and HDF cells^30^. The major reason might be caused by a limited release of PhIP molecules from the particulate PhIP in comparison with the dissolved PhIP in DMSO.

In this study, cytotoxicity of L02, HepG-2, A549 and HelF cells incubated with PhIP, MeIQx and TDA self-made aerosol particles under low concentration from 0.5 to 4 μg/mL were investigated. To our knowledge, this was the first study to comprehensively evaluate low concentration aerosol particles’ exposure risks of heterocyclic aromatic amines and aldehydes.

In the literature, PhIP and MeIQx were evaluated as potent DNA damaging agents through MTT cell viability assay and the single cell gel electrophoretic assay in HepG-2 cells extraction from home cooked and commercially available food sources^36^. Depending on the exposure concentration/time, Stampar showed that PhIP’s DMSO solution at 400 μM (89.7 μg/mL) after 72 h exposure significantly decreased cell viability in HepG-2 spheroids by approximately 24% due to the DNA damage^35^. Cooking areosol contained significant amounts of MeIQx, but with unclear association between MeIQx solution and lung cancer by culture of TA98^24^. MeIQx was reported to induce liver tumors in mice fed a diet containing 0.06% MeIQx for 84 weeks^26^. A previous study showed that exposure to a sublethal dose of 1 μM (0.15 μg/mL) TDA solution for 45 days significantly induced the proliferation of human bronchial epithelial cells (BEAS-2B), which is considered the early stage of COF-associated lung carcinogenesis^29^. TDA (7.6–190 μg/mL) was reported to be associated with lung cancer and to involve inhibitors of apoptosis proteins in cell survival and proliferation of A549 lung cancer cells in vitro^37^. Our CCK-8 assay results first confirmed that the cytotoxicity of low dose PhIP aerosol particles to L02 among L02, HepG-2, A549 and HelF cells, while low dose of MeIQx and TDA aerosols brought little cell apoptosis. Flow cytometry results further manifested the adverse effect of PhIP to L02 cell, which were almost identical to those of the CCK-8 results.

ROS formation most likely occurred during the autoxidation of both polyunsaturated and monounsaturated fatty acids^38^. Sunflower and rapeseed oils produced the highest ROS concentrations (80.48 and 71.75 nmol/(g cooking material and oil)·h, respectively) among five types of edible oil^38^. A recent study proved that only ROS concentrations coming from certain types of chinese cooking exhibited consistency with cell viability, and showed significant correlations with genetic damage and expression in human bronchial epithelial cells^38^. PhIP might be generating ROS by more than one mechanism, which would induce DNA damage and cell toxicity^39^. ROS was generated from MeIQx and the ROS might be involved in mutagenesis^40^. An early study demonstrated that TDA can lead to cytotoxicity and oxidative DNA damage through the induction of ROS at concentrations from 50 to 200 μM in A549 cells^41^. So, PhIP and MeIQx were widely accepted the cell damage through ROS, in combination with the results from confocal imaging of HepG-2 and L02 cells stained with DCF-DA.

A previous investigation exhibited that COFs exposure to female rats can affect follicular structure through follicular cell damage and apoptosis in ovaries, while no pathological abnormality was observed in the control rat^42^. The exposed rats for 30 and 60 days to cotton oil fumes showed a significant increase in the lung and liver malondialdehyde levels which accompanied with a significant decrease in glutathione content. DNA change was clear in the lungs of rats after cotton oil fumes exposure in previous report^43^. The result suggested that PhIP origining from COFs was a direct cause of toxicity to the liver and lung of mice in a dose-dependent manner under increased dose incubation to a high level or exposure to longer times. The data in this study suggested that the low-dose biological adverse effects assessment in vitro and in vivo was more representative exposure to heterocyclic aromatic amines and aldehydes compared to previous studies with high dose of their solution.

## Conclusion

In summary, by employing in vitro and in vivo experiments on cancer and healthy lung and liver cells, and mice under low-dose exposure to PhIP, MeIQx and TDA aerosol particles, their biological adverse effects were systematically assessed. A significant conclusion was that PhIP, MeIQx and TDA aerosol particles had negligible cytotoxicity to cancer cells (A549, HepG2), which exhibited cytotoxicity to normal healthy cells (HelF, L02) under 0.5–4 μg/mL low-dose exposure based on the reactive oxygen species (ROS) mechanism. Furthermore, body weights and H&E-stained images of main organs (heart, liver, spleen, lung, kidney) from mouse groups after PhIP exposure demonstrated that PhIP caused significant damage to the lung and liver. This was especially important considering the direct proof of COF damage even at low dose exposure to HAAs and aldehydes.

## Materials and methods

### Materials

Amine 2-amino-1-methyl-6-phenylimidazo[4,5-b] pyridine (PhIP) and 3,8-dimethylammidazo[4,5-f] quinoxalin-2-amine (MeIQx) were purchased from Sinopharm Chemical Reagent Co., Ltd. Trans, trans-2,4-decadienal (TDA, > 90%) was purchased from Shanghai Yuanye Bio-Technology Co., Ltd. 2',7'-dichlorodihydrofluorescein diacetate was purchased from Aladdin. Cell Counting Kit-8 (CCK-8) was obtained from Dojindo China Co., Ltd. Dulbecco’s modified Eagle’s medium (DMEM), fetal bovine serum (FBS), and phosphate buffer solution (PBS) were all purchased from Beijing Solarbio Science and Technology Co., Ltd. Deionized water was used in all experiments. All reagents were used as received without additional purification.

### Fabrication for aerosol particles and their characterization

A 1 mL DMSO solution containing 10 mg PhIP, MeIQx and TDA was prepared for mother liquid, seperately. The above solutions were injected into a compressor nebulizer (InnoSpire Deluxe, Philips). Thus, particulate PhIP, MeIQx and TDA aerosols were obtained by spraying out their DMSO solution, respectively, which were diluted with ethanol and PBS for further transmission electron microscopy (TEM) and dynamic light scattering (DLS) measurements. The morphology of their aerosol particles was observed on a JEM-2100F high-resolution TEM operating at an accelerating voltage of 200 kV. Their zeta potential and size distribution were conducted with photon correlation spectroscopy (PCS, Nanosizer Nano ZS, MALVERN Instruments).

### In vitro cytotoxicity of aerosols

100 μL, 10 mg/ml DMSO solution containing PhIP, MeIQx and TDA was diluted to 10 mL DMEM (containing 1% penicillin–streptomycin and 10% FBS) to obtain 100 μg/mL solution, seperately, named solution A. 10 mL solution A was injected into a compressor nebulizer to obtain PhIP, MeIQx and TDA aerosols by spraying out solution A. Then the collected aerosols were diluted with DMEM to different concentrations (0.5, 1, 2, 4, 5, 10, 25, 50 μg/mL). To evaluate the cytotoxicity performance of PhIP, MeIQx and TDA aerosols, a standard CCK-8 cell assay was used with different cell lines (HepG-2, L02, HelF and A549 cells), and DMEM containing 1% penicillin–streptomycin and 10% FBS was used as the culture medium. First, the cells were seeded in 96-well plates (8000 cells/well) and incubated under a humidified atmosphere (5% CO_2_, 37 °C) for 24 h. Then, then 200 µL DMEM solution containing PhIP, MeIQx and TDA aerosols with required different concentrations varied from 0.5 to 50 μg/mL were co-incubated with cells for an additional 24 h, 48 h and 72 h. Subsequently, 100 µL of culture medium containing CCK-8 (10%) was incubated with the cells for 1 h. Finally, the cell viability was calculated as the ratio of the absorbance of the wells. The absorbance at 450 nm was measured by Thermo Multiskan FC. To assess apoptosis assay on HepG2 and L02 cells after 24 h exposure to PhIP, MeIQx and TDA aerosols with 4 μg/mL (200 µL), according to the manufacturer's protocols, apoptosis was measured with double stained with Annexin-V-FITC apoptosis detection kit and propidium (PI) (eBioscience, CA, USA) and analyzed using a flow cytometer (FACSVerse, BD Biosciences, USA).

### In vitro imaging

HepG-2 and L02 cells at a density of 10,000 cells were seeded into a plate for 24 h at 37 °C and then incubated with PhIP, MeIQx and TDA aerosol concentrations 4 μg/mL (200 µL) for 6 h in a humidified atmosphere. Fresh cells were prefixed in a stationary liquid of 3% glutaraldehyde–paraformaldehyde and were then postfixed with 1% osmium tetroxide and 1.5% potassium ferrocyanide (Sigma). The samples were dehydrated with increasing concentrations of alcohol and finally with acetone. The specimens were embedded in Araldite resin 618 and examined with TEM (HT7700). After washing with PBS three times, the cells were stained with dichlorodihydrofluorescein diacetate (DCF-DA) and documented by a Nikon laser confocal superresolution fluorescence microscope (N-C2-SIM) to evaluate ROS.

### In vivo toxic performance of PhIP aerosols

100 μL, 10 mg/ml DMSO solution containing PhIP was diluted to 10 mL PBS to obtain a 100 μg/mL solution. Then, 10 mL of 10, 20, 50 μg/mL PhIP solution were obtained by diluted the above mother liquid. Male nude Balb/c mice (6 weeks old) were purchased from the Beijing Laboratory Animal Center, Chinese Academy of Sciences (SLACCAS). All animal procedures were conducted according to institutional regulations regarding animal use and care, as approved by the Model Animal Research Center of Institute of Process Engineering, Chinese Academy of Sciences. First, the mice were divided into five groups, and each group contained three mice. The groups were labeled PBS, A, B, C and D. The 10 mL different concentration (10, 20, 50, 100 μg/mL) PhIP solution dispersion in PBS was nebulized nearlly 30 min, and was inhaled by the A, B, C and D mice groups by compressor nebulizer every day, respectively, while the PBS (control) group being administered PBS only. Over the next 30 days, mouse weights in the five groups were measured every two days. After 30 days, all mice were euthanized, and body organs were removed for histological examination through Nikon laser confocal super resolution fluorescence microscope (N-C2-SIM).

### Ethics approval

The study is reported in accordance with ARRIVE guidelines (PLoS Bio 8(6), e1000412,2010). Institute of Process Engineering, Chinese Academy of Sciences owns the license plate (SYXK2019-0004). All animal procedures were conducted according to institutional regulations regarding animal use and care, as approved by the Model Animal Research Center of Institute of Process Engineering, Chinese Academy of Sciences.

## Date availability

The datasets generated and analyzed during the current study are available from the corresponding author on reasonable request.
